# *Escherichia coli* and *Enterobacteriaceae* Counts, Virulence Gene Profile, Antimicrobial Resistance, and Biofilm Formation Capacity during Pig Slaughter Stages

**DOI:** 10.3390/life14101261

**Published:** 2024-10-03

**Authors:** Madalena Maria Saldanha Coelho, Emilia Fernanda Agostinho Davanzo, Rebecca Lavarini dos Santos, Virgílio Hipólito de Lemos Castro, Hayanna Maria Boaventura da Costa, Bruno Stéfano Lima Dallago, Simone Perecmanis, Angela Patrícia Santana

**Affiliations:** Faculty of Agronomy and Veterinary Medicine, University of Brasília (UnB), Federal District, Brasília 70910-900, Brazil; madalena.coelho@seagri.df.gov.br (M.M.S.C.); emiliadavanzo@unb.br (E.F.A.D.); virgilio.castro@edu.se.df.gov.br (V.H.d.L.C.); hayanna.maria@unb.br (H.M.B.d.C.); dallago@unb.br (B.S.L.D.); perecmaniss@unb.br (S.P.); patvet@unb.br (A.P.S.)

**Keywords:** swine, *Escherichia coli*, biofilms, antimicrobial multiresistance

## Abstract

This study aimed to count *Enterobacteriaceae* and *Escherichia coli* in different locations on pig carcasses (shank, loin, abdomen, shoulder, and jowl) from two slaughterhouses (A and B) between September 2019 and July 2021 during different slaughter stages (after bleeding, after passing through the epilator machine, after manual toileting in the dirty area, before and after evisceration, and after the final washing), as well as verify antimicrobial resistance and biofilm formation capacity. The main points of *Enterobacteriaceae* and *E. coli* contamination were identified in the two slaughterhouses through three collections. The stages with the highest counts were post-bleeding and evisceration in both slaughterhouses and after manual toileting in slaughterhouse B in the first collection. Most *E. coli* isolates were resistant to multiple antimicrobials, with higher resistance frequencies to amoxicillin, ampicillin, chloramphenicol, sulfonamides, and streptomycin. The virulence genes *eae*, *stx1*, and *stx2* were also detected. Three isolates had all three genes and exhibited resistance to at least six antimicrobial classes (β-lactams, macrolides, aminoglycosides, sulfonamides, amphenicols, and quinolones). *E. coli* isolates also showed a high frequency of strains with moderate and strong in vitro biofilm-forming capacity. This is the first study to characterize microbial contamination by pig slaughter stage in the Federal District region, demonstrating the critical points for hygienic production. *E. coli* was isolated from the surface of pig carcasses, as well as the virulence genes *stx1*, *stx2*, and *eae* were detected. The multi-antimicrobial resistant isolates also had a moderate-to-strong biofilm formation capacity, thus demonstrating risks to public health.

## 1. Introduction

Brazilian pork production in 2020 was approximately 4.5 million tons, and more than 1 million tons were exported, with the domestic market using approximately 3 million tons and 16 kg consumption per inhabitant [1]. According to the Brazilian Association of Animal Proteins [1], Brazil is the fourth largest pork exporter worldwide, and the Federal District (DF) is responsible for 0.4% of Brazilian pig breeding, with approximately 200 thousand porcine in 2020 [2].

Production process intensification was attributed to meat market demands, which increased the densities of pig breeding products, causing sanitary pressure in the production system. This increases concern about microorganisms in the production chain and their effects [3,4,5,6].

Microbiological contamination of food is a global problem [7] and responsible for two-thirds of foodborne disease outbreaks. Animals are the main microbial load carriers in products of animal origin due to contamination, both from the gastrointestinal contents and processing environment areas [8,9].

The microorganisms commonly involved in outbreaks of foodborne illnesses (DTAs) include *Salmonella*, *Staphylococcus aureus*, *Campylobacter*, *Listeria monocytogenes*, and *Escherichia coli* [9,10,11]. Brazilian legislation requires the microbiological control of cattle and pig carcasses in refrigerated slaughterhouses to evaluate process hygiene and reduce the prevalence of these pathogenic agents [12]. Furthermore, the *Enterobacteriaceae* family count was included in the evaluation of carcasses after the final wash in slaughterhouses before the cooling chamber to evaluate the hygienic conditions of slaughter, following the international guides of microorganism analyses [12,13].

*Enterobacteriaceae* contagion was analyzed as an indicator of hygiene conditions at slaughter since they can be found in the environment and slaughtered animal intestines and can also indicate food contamination directly or indirectly by fecal content or environmental factors [14,15]. *E. coli* is relevant among those of fecal origin [15] as some strains, such as uremic–hemolytic Shiga toxin-producing *E. coli* (STEC) [16], enteropathogenic *E. coli* (EPEC), enteroaggregative *E. coli* (EAgEC), enteroinvasive *E. coli* (EIEC), and enterohemorrhagic *E. coli* (EHEC) [9], can cause foodborne diseases.

Few studies have evaluated the microbial counts at each stage of pig slaughter in Brazil [17], with most microorganism studies focusing on just one part of the carcass or industrial processing environment [14,18,19,20,21,22,23]. The *Enterobacteriaceae* and *E. coli* count during the technological processing stages of pig slaughter in the Federal District or in various pig carcass points/parts has not been studied. According to Pinto [24] and Costa et al. [25], it has been used to carry out risk analysis and identify critical control points within pig slaughter, aiming to monitor and change whenever necessary, and is a way to ensure food safety [26].

Therefore, this study aimed to evaluate the *Enterobacteriaceae* and *E. coli* counts at different carcass points/parts and at different pig slaughter stages in refrigerated slaughterhouses located in the Federal District region to study and identify the *stx1*, *stx2*, and *eae* genes in *E. coli* isolates, as well as evaluate their biofilm formation capacity and antimicrobial resistance.

## 2. Materials and Methods

### 2.1. Sample Origin

This study was conducted in two refrigerated pig slaughterhouses (A and B) located in the Federal District region, under official inspection, between September 2019 and July 2021. The industries agreed to participate spontaneously and voluntarily in this study, and the pigs did not come from integrated farms. Three collections were conducted, and a carcass was randomly selected and monitored at each stage of the slaughter process to swab defined points on the carcass surface. At each visit, a collection was carried out, with one collection/visit at cold storage slaughterhouse A and two visits at cold storage slaughterhouse B.

The samples were obtained from sterile cotton swabs (Absorve^®^ Cotia, São Paulo, Brazil) used with sterilized polyamide and stainless-steel molds, respectively, with an area of 9 cm^2^ in the collection from the refrigerated slaughterhouse A and 100 cm^2^ in the collection of the refrigerated slaughterhouse B. The swabs were previously moistened in sterile 0.1% peptone water and, after scrubbing, were placed individually in tubes containing the transport solution as described by Corbellini et al. [14] and Davanzo et al. [27].

Five points/locations were selected for the pig carcass, and six points/stages were selected for the technological processing of pig slaughter. The points/locations chosen to collect swab samples followed the protocols described by Matsubara [28], the MAPA Animal Product Sample Collection Manual [29], and Cê [30].

Swabs were collected from the shank, loin, abdomen, shoulder, and jowl of a randomly selected carcass at selected stages during the technological process of slaughter: after bleeding, after depilation, after manual depilation (toilet), before and after evisceration, after washing the carcass in the clean area, and before moving the carcass into the cooling chamber. Individual sterile swabs (Absorve^®^) were used at each point for each stage, making 30 swab samples for each slaughterhouse, with 90 samples for both establishments.

### 2.2. Enterobacteriaceae and E. coli Count

*Enterobacteriaceae* and *E. coli* were counted according to the method AOAC method (Association of Official Analytical Chemists) [31] using 3MTM Petrifilm TM *Enterobacteriaceae* Count Plate (EB) and Petrifilm TM 3MTM *E. coli*/Coliform Count Plate (EC), respectively. The swabs were homogenized with 1 mL transport solution, inoculated in both EB and EC plates, and incubated at 35 ± 1 °C for 24 h. The counting methodology on Violet Red Bile Glucose agar (Acumedia^®^) was used, in accordance with the ABNT NBR ISO 21528-2:2020 standard [32], to count *Enterobacteriaceae*. After inoculating the samples, they were incubated at 35 °C for 24 h. The counts for each analysis were converted into logarithmic values.

### 2.3. E. coli Isolation

Regarding *E. coli* isolation by stage of the technological processing of slaughter and location of the carcass in slaughterhouses A on 1st visit and B on 2nd visit, the swabs collected from slaughterhouses A and B were seeded directly on eosin methylene blue (EMB) agar plates (KASVI^,^Weissópolis, Paraná, Brasil) and incubated at 37 °C for 24 h. Colonies considered typical were subjected to biochemical tests as described by Silva et al. [31] to confirm the presence of microorganisms.

### 2.4. Isolate Antibiogram

A total of 52 *E. coli* samples were subjected to an antibiogram using the disk diffusion technique standardized by the Clinical and Laboratory Standards Institute manual [33]. To perform the antibiogram, five disks (SENSIDISC, DME, Araçatuba, São Paulo, Brazil) were placed on the agar surface. The following pharmacological bases were used: Quinolones: nalidixic acid (NAL: 30 μg), ciprofloxacin (CIP: 5 μg); β-Lactams: amoxicillin (AMX: 10 μg), ampicillin (AMP: 10 μg); Cephalosporins: cefazolin (CFZ: 30 μg), ceftazidime (CAZ; 30 μg); Amphenicols: chloramphenicol (CLO: 30 μg); Tetracyclines: tetracycline (TET: 30 μg), doxycycline (DOX: 30 μg); Macrolides: erythromycin (ERI: 15 μg); Aminoglycosides: streptomycin (EST: 10 μg), gentamicin (GEN: 10 μg); Sulfonamide: sulfonamides (SUL: 300 μg). The results followed the CLSI M100-ED31:2021 standard [34].

### 2.5. E. coli Virulence Gene Detection

Polymerase chain reaction (PCR) was used to investigate the presence of Shiga toxin gene types 1 and 2 (*Stx1* and *Stx2*) and *eae* genes, as described by China et al. [35]. DNA was extracted through boiling method. Then, 5 μL product containing approximately 5 ng/μL total DNA estimated, as sample, was amplified in a 25 μL reaction mixture containing 1× buffer (200 mM Tris-HCl, pH 8.4, 500 mM KCl—PHT^®^), 2.0 mM dNTPs, 2 U taq DNA polymerase (PHT^®^), 2 mM phosphate deoxyribonucleotides (Invitrogen^®^,Waltham, MA, USA), 3 mM MgCl_2_ (PHT^®^), and 1 μM primers on a MyCycler thermocycler (BioRad^®^,Hercules, CA, USA. The primers, annealing temperatures, and expected fragment size are described in Table 1.

The products were visualized in 1.5% agarose gel (Invitrogen^®^ Waltham, MA, USA) stained with 0.5 μg/mL ethidium bromide on a transilluminator device (Imagequant, Gelifesciences^®^) and imaged (Major Science UV^®^, Saratoga, CA, USA). Positive controls for *stx1*, *stx2*, and *eae* were obtained from the bacteriolytic library of the Food Microbiology Laboratory/FAV/UnB.

### 2.6. In Vitro Biofilm Formation Capacity Evaluation

The in vitro biofilm formation capacity of *E. coli* isolates was evaluated as described by Djordjevic et al. [36] and adapted by Borges et al. [37]. Each isolate analysis was performed in triplicate at 37, 25, and 10 °C for 24, 48, or 72 h. The analyses at 37 °C and 25 °C were carried out in a bacteriological incubator (Quimis^®^ and Fanem^®^, respectively), while those at 10 °C were carried out in a refrigerator (Eletrolux^®^, Stockholm, Sweden)

After the incubation, the inocula were removed using a pipette for subsequent washing with sterile 0.9% saline solution three times. The plates were then dried at room temperature, treated with 200 µL methanol PA (Vetec^®^—Sigma-Aldrich, St. Louis, MO, USA) for 15 min, and then dried for 15 min at room temperature after removing the methanol by inverting the plates. Thereafter, the plates were treated with 200 µL 1% Hucker’s crystal violet (Newsprov^®^) to stain the isolate adhesion areas for 10 min, washed with running water, and dried at room temperature. Subsequently, 200 µL 33% acetic acid (J. T. Baker^®^) was added to each sample, and the absorbance at 490 nm was measured using an ELISA reader (Biotek^®^Elx800).

The result was considered as the reading average of three samples of each isolate and compared to that of the negative control (Don). The mean absorbance obtained from triplicate readings was used to determine the final optical density of each strain (ODf), which was compared with that of the negative control (ODn). The isolates were categorized into non-biofilm-forming isolates (NF) when ODf ≤ ODn, weakly biofilm-forming when ODn < ODf ≤ 2× ODn, moderate biofilm-forming when 2× ODn < ODf ≤ 4× ODn, or strong biofilm-forming when 4× ODn < ODf [38].

Statistical analyses were performed to verify the biofilm formation ability of each isolate by correspondence analysis, chi-square test, and Fisher’s test when relevant, thus checking the greater or lesser capacity for formation between temperatures, incubation durations, and the collection sites at slaughter and carcass. All analyses were performed using SAS^®^ software (v.9.4, Cary, NC, USA) at a significance level of 5%.

## 3. Results and Discussion

### 3.1. Enterobacteriaceae and E. coli Count

The *Enterobacteriaceae* and *E. coli* counts obtained from each pig carcass location/point, as well as each pig slaughter stage, are described in Table 2, Table 3, Table 4, Table 5, Table 6 and Table 7.

**Figure 1:**
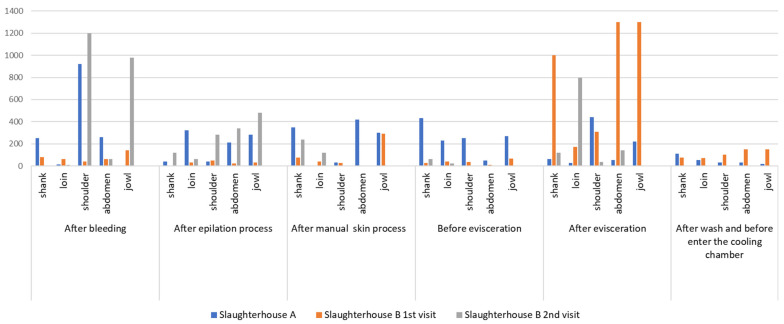
Pig carcass count of *Enterobacteriaceae* (CFU/cm^2^) in slaughterhouses A and B (1st and 2nd visits) by stage of technological processing of pig slaughter and by location/point on the carcass.

**Figure 2:**
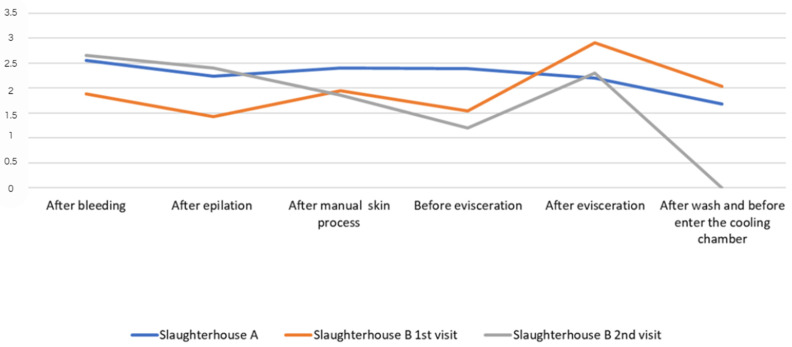
General average of *Enterobacteriaceae* counts from all parts of the pig carcass, converted into log CFU/cm^2^, from each stage of the technological processing of slaughter in slaughterhouses A (1st visit) and B (1st and 2nd visits).

The results obtained for *Enterobacteriaceae* counts (CFU/cm^2^) for pig carcass parts from slaughterhouses A and B for each visit and at each slaughter stage are described in Figure 1 and Figure 2.

In general, few studies in Brazil have focused on *Enterobacteriaceae* count in pig carcasses, as can be seen in the review conducted by Viltrop et al. [17]. No published studies have reported *Enterobacteriaceae* count by carcass location/point and at different slaughter stages in the Federal District. Microorganism analysis was established in 2018 through Normative Instruction number 60 from the Ministry of Agriculture, Livestock, and Supply [12], which may explain the absence of related work. Figure 1 and Figure 2 show a similar profile for *Enterobacteriaceae* counts/cm^2^ and average counts converted into log CFU/cm^2^ for all carcass parts at each slaughter stage in slaughterhouses A and B, with higher peaks due to higher counts after bleeding and evisceration, which corroborates the results reported by Wheatley et al. [39], describing greater contamination in the jowl and abdomen areas after evisceration. The high count in the shoulder and jowl regions in the post-bleeding stage in both slaughterhouses corroborates the analysis by Bolton et al. [40], in which a higher bacterial count may be associated with contact of the pig carcass with the slaughterhouse floor or other instruments present in the environment at this stage.

In the analysis of the pig slaughter technology flowchart, a higher count of *Enterobacteriaceae* count/cm^2^ was found in slaughterhouse A, in the post-bleeding stage, and in the general carcass average for refrigerated slaughterhouses A and B (second collection), whose log CFU/cm^2^ values were 2.55 and 2.65, respectively. Subsequently, the count decreased after hair removal and manual skinning, increased after the post-evisceration stage, and decreased after washing and before entering the cooling chamber. These findings demonstrate a reduction in the count of microorganisms after bleeding, probably due to the action of the scalding process, depilation stage, and final washing of the carcasses, which are stages provided by Ordinance 711 of 1995 of the Ministry of Agriculture, Livestock, and Supply [41], which deals with procedural standards for pig slaughter and inspection. The increase in counts observed in this study in the post-evisceration stage corroborates the reports by Bolton et al. [40] regarding the high count of aerobic microorganisms at this stage. According to the authors, the higher count may be related to the spread of microorganisms due to the nature of the evisceration procedure, as well as to equipment/utensils contaminated at this stage and to the ambient air due to the release of fomites.

The average *Enterobacteriaceae* count, which was obtained from the sum of the counts of all pig carcass points/parts for each slaughter stage and expressed in log CFU/cm^2^, was 2.5 log CFU/cm^2^ after bleeding for slaughterhouse A in the first collection, 1.88 log CFU/cm^2^ for slaughterhouse B in the first collection, and 2.6 log CFU/cm^2^ in the second collection (Table 2, Table 3, Table 4, Table 5, Table 6 and Table 7 and Figure 2). These values were lower than those obtained by Corbellini et al. [14], who reported 3.5 log CFU/cm^2^ in pig carcasses in slaughterhouses located in southern Brazil. Although the results of the present study were obtained from a single carcass for each visit to slaughterhouses A and B, the results were close to those verified by Montes et al. [42] in pig carcasses from the western region of Santa Catarina, Brazil (2.37 log CFU/cm^2^ in the bleeding stage). However, the results of the average carcass in log CFU/cm^2^ in the stage after bleeding, with values obtained of 2.55 log CFU/cm^2^ (A first collection), 1.88 log CFU/cm^2^ (B first collection) and 2.65 log CFU/cm^2^ (B second collection), were higher than those obtained by Cê (1.83 log CFU/cm^2^) [30], in pig carcasses from slaughterhouses located in Santa Catarina, Brazil.

Unlike the results of other studies describing recontamination of carcasses during epilation due to the difficulty of sanitizing the equipment and fecal contamination [40,43,44], in this study, there was no large increase in the logarithmic *Enterobacteriaceae* count after the epilation stage. Comparing the stages before scalding (after bleeding) and after epilation (after scalding), the logarithmic *Enterobacteriaceae* count decreased to 0.31 log CFU/cm^2^ in the first collection from slaughterhouse A, 0.45 log CFU/cm^2^ in the first collection from slaughterhouse B, and 0.25 log CFU/cm^2^ in the second collection from slaughterhouse B. The reduction in *Enterobacteriaceae* counts after hair removal (Figure 2) can be explained by scalding followed by epilation and the absence of recontamination in the epilation machine. Thus, these results corroborate those of Spescha et al. [43], Wheatley et al. [39], and Nastasijevic et al. [45], who demonstrated that scalding pig carcasses reduced bacterial contamination.

In both refrigerated Slaughterhouse A and the first collection from Slaughterhouse B, the skin processes were made in the dirty area, according to the classification given by Ordinance No. 711 of 1995 of the Ministry of Agriculture, Livestock, and Supply [41]. The procedure was manually performed on a table, under which the hair remained, and with high humidity, which may have contributed to the increased microbial count at this stage, even though there was a reduction in the previous stage of scalding. However, there was a reduction in 0.54 log CFU/ cm^2^ for slaughterhouse B in the second collection. The logarithmic results of the overall carcass average (Table 4) from the manual toilet stage, from the first collections from slaughterhouses A and B (2.4 and 1.94 log CFU/cm^2^ respectively), increased from those from the previous stage (hair removal). Comparing the microbial count at the manual toilet stage in this study with those at the mechanized toilet stage verified by Corbellini et al. [14], in the dirty area, the results are similar to those observed by the authors, including that at the previous steps, such as the post-epilation step. However, these results differed from those observed by Zwirzitz et al. [46], who did not detect *Enterobacteriaceae* at the skin stage. The results of this study were also different from those obtained by Cê [30] and Rodrigues et al. [22], who did not detect *Enterobacteriaceae* or only detected a low count at the skin process stage. A possible cause may be related to the knives used by handlers at this stage, which pass through areas close to the oral cavity and perianal region. This, in turn, is related to inadequate ways of carrying out this procedure, thus characterizing cross-contamination, although Buncic and Sofos [47] and Wheatley et al. [39] also verified contamination in the practice of mechanized toileting.

Evaluating the general logarithmic results of the carcasses (Table 2, Table 3, Table 4, Table 5, Table 6 and Table 7 and Figure 1 and Figure 2) before and after evisceration in slaughterhouse B indicated an increase of more than 1 log CFU/cm^2^ in the two collections (2.91 and 2.3 log CFU/cm^2^ from the first and second collections, respectively). In relation to the results per point/location on the carcass, the shoulder (4.4 × 10^2^ CFU/cm^2^) presented the highest *Enterobacteriaceae* count after evisceration in slaughterhouse A in the first collection. In cold slaughterhouse B, the belly and jowls presented a count of 1.3 × 10^3^ CFU/cm^2^ in the first collection, and the loin had the highest count (8.0 × 10^2^ CFU/cm^2^) in the second collection. Wheatley et al. [39] found significant increases in counts in the jowls and abdominal area after evisceration, as seen in this study. According to them, evisceration is frequently the biggest source of contamination in pig slaughter since even healthy animals can be carriers of pathogenic and opportunistic microorganisms due to changes in the permeability of the intestinal wall as a result of stress during slaughter. These microorganisms can penetrate other tissues, as the handler can be the vehicle for transmission to other areas, leading to increased counts [48], thus justifying the results of this study.

MAPA Normative Instruction No. 60, of 20 December 2018 [12] establishes microbiological count standards of *Enterobacteriaceae*, in log CFU/cm^2^, for pork carcasses before cooling as acceptable, with the standard being lower than 2.0 log CFUcm^2^, intermediate standard with counts between 2.0 and 3.0 log CFU/cm^2^, and unacceptable standard with counts above 3.0 log CFU/cm^2^. In this study, the carcasses from slaughterhouse A in the first collection and those from slaughterhouse B in the second collection could be classified as acceptable after washing and before cooling. The carcass from the first collection from Slaughterhouse B was classified as intermediate, as it presented a logarithmic count of 2.04 log CFU/cm^2^.

### 3.2. E. coli Counts

**Figure 3:**
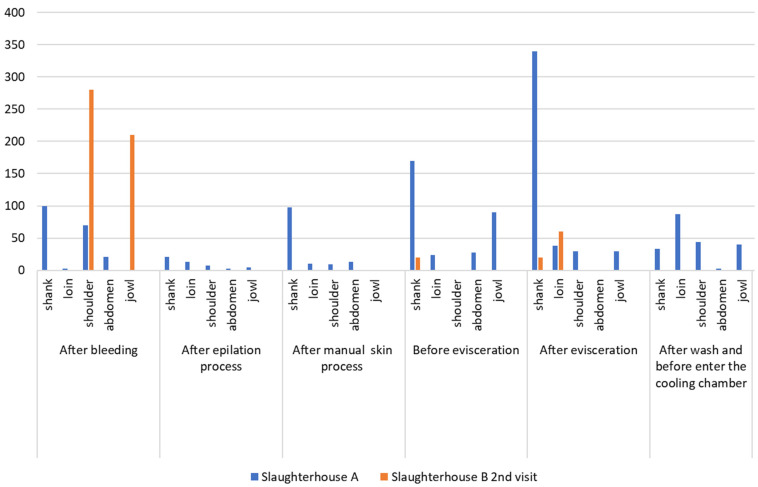
Count of *E. coli*/cm^2^ at different points on the pig carcass and at different stages of the slaughter process in slaughterhouses A and B (2nd collection).

**Figure 4:**
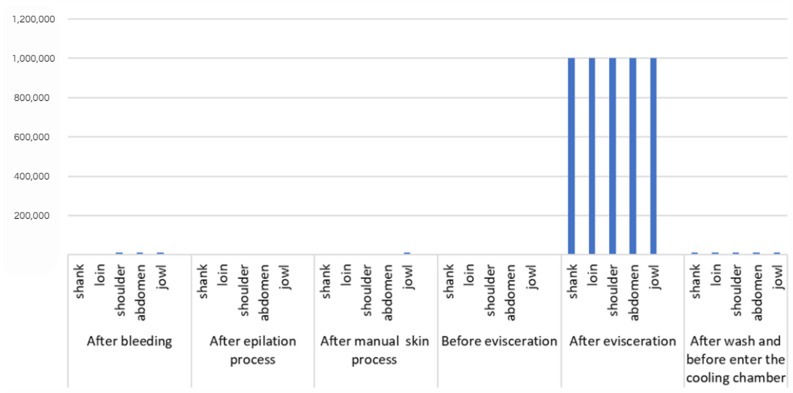
Count of *E. coli*/cm^2^ at different points on the pig carcass and at different stages of the slaughter process in slaughterhouses A and B (1st collection).

**Figure 5:**
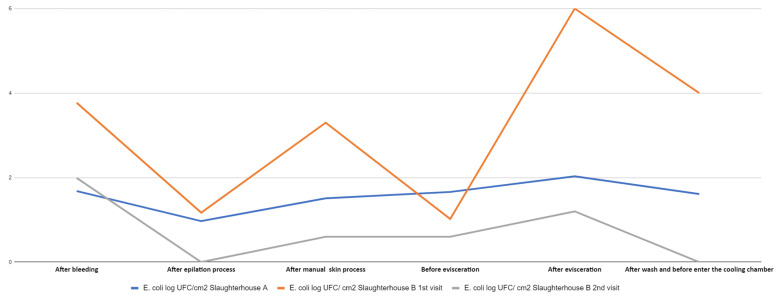
General average of *E. coli* counts in log CFU/cm^2^ in different parts of the pig carcass from slaughterhouses A (1st collection) and B (1st and 2nd collection) at different stages of the slaughter process.

Figure 3 and Figure 4 present the *E. coli* counts (CFU/cm^2^) from pig carcass points/parts, which revealed an increased count in the stages after bleeding and evisceration; the *E. coli* count for all carcass parts in the post-evisceration stage in slaughterhouse B, in the first collection cm^2^, which decreased to 10^4^ CFU/cm^2^ after washing the carcass and the jowl. Figure 5 shows the average *E. coli* counts converted into logarithmic values (log CFU/cm^2^) from slaughterhouses A and B and for each slaughter stage.

According to Ghafir et al. [49], *E. coli* and *Enterobacteriaceae* counts are normally correlated and used in slaughterhouses as environmental and fecal contamination indicators; both appear throughout the technological processing of slaughter in this study, indicating contamination. High counts were observed, with a value of 10^4^ CFU/cm^2^ in the jowl, shoulder, and abdominal regions, at the post-bleeding stage, in the first collection from slaughterhouse B.

In this study, *E. coli* counts were also higher in the stages after bleeding (overall average carcass count of 1.68 log CFU/cm^2^ in slaughterhouse A in the first collection, 3.77 log CFU/cm^2^ in slaughterhouse B in the first collection, and 1.99 log CFU/cm^2^ in slaughterhouse B in the second collection) and after evisceration (average counts of 2.03 log CFU/cm^2^ in slaughterhouse A in the first collection, 6.0 log CFU/cm^2^ in slaughterhouse B in the first collection, and 1.2 log CFU/cm^2^ in slaughterhouse B in the second collection). The respective peak values are shown in Figure 5, which presents the average *E. coli* counts converted into log CFU/cm^2^ from all carcass points/locations by stage and sequence of the technological processing of slaughter. The points/locations on the carcass with the highest *E. coli* counts were the jowls, shoulder, and abdomen in the stages after bleeding, skinning, evisceration, and before the cooling stage in slaughterhouse B in the first collection. Slaughterhouse A had the highest count, which remained until the post-evisceration stage. The results obtained in this study differed from the *E. coli* prevalence on pig carcass surfaces in the southern region of Brazil observed by Machado et al. [50], where the jowls had high *E. coli* counts.

According to Rivas et al. [51], final washing did not reduce the microbial count in the carcass. However, this study verified decreased *E. coli* counts in carcasses after final washing than those after the previous evisceration stage. Comparing the results of these two stages, the average *E. coli* count after evisceration (2.03 log CFU/cm^2^) decreased after washing (1.61 log CFU/cm^2^) in slaughterhouse A. In this study, the average *E. coli* count after evisceration also decreased after washing in the first (6.0 and 4.0 log CFU/cm^2^, respectively) and second (1.20 log CFU/cm^2^ and undetected, respectively) collections from slaughterhouse B. Even though residual chlorine content was not measured in the washing water in this study, Brazilian legislation determines a 0.2 to 2.0 ppm chlorine limit in drinkable water [13]. Therefore, the presence of chlorine may be a possible justification for the superficial microbial reduction or absence of growth after washing the carcasses observed in this study.

The *E. coli* count on the pig carcass surface obtained in this study do not provide parameters for Brazilian legislation, which does not consider counting *E. coli* in pig carcasses for microbiological control. In Brazil, *Enterobacteriaceae* count and the presence or absence of *Salmonella* spp. are used, following the model established by the European Union, which also counts aerobic colonies to evaluate the hygiene of the meat production process [52]. However, the *E. coli* count can be considered as a quality control indicator since the profiles verified for this microorganism were similar to those of *Enterobacteriaceae*.

Although *Enterobacteriaceae* and *E. coli* counts were carried out on a few carcasses, which in turn may not show the overall real profile of the national industry, the results showed the same count profile, in which the peaks with higher values coincided with the same steps between the two analyses, which in turn can contribute to improvements in the pig slaughter flowchart in industries. Furthermore, the counts in different carcass parts showed similarities in places with the highest counts between the two analyses, which can contribute to good manufacturing practices in this type of establishment, making it the first in the region and country.

### 3.3. E. coli Isolates

Twenty-three *E. coli* isolates were obtained in the first collection from slaughterhouse A, and 29 isolates in the second collection from slaughterhouse B, for a total of 52 isolates (Table 8). The virulence genes and in vitro biofilm formation capacity of the isolates were studied.

### 3.4. Antibiogram of E. coli Isolates

The antibiograms and frequency results for the 52 *E. coli* isolates from slaughterhouses A and B are presented in Table 9 and Table 10. The drugs with the highest percentage of resistance detected in this study were beta-lactams (amoxicillin and ampicillin, 88.5% resistant isolates), followed by sulfonamide (84.61%), chloramphenicol (82.7%), and streptomycin (80%). According to the CLSI [34], the antibiogram results for ampicillin can be used to predict the results for amoxicillin, which explains the same proportion of resistance to both antimicrobials in this study.

According to the definition of multidrug resistance stipulated by Magiorakos et al. [53], 49 *E. coli* isolates (94.23%) were considered multidrug-resistant, presenting resistance to three or more classes of antimicrobials. Similar results were obtained by Mangal et al. [51], in an evaluation of pork from India and identified 100% resistance to ampicillin in 41 *E. coli* isolates. Similar to the findings of this study, Pissetti et al. [54] detected a multidrug resistance profile in 71.5% strains from swine carcasses in Santa Catarina, Brazil, with 97.8%, 86.4%, 85.1%, and 81.1% strains being resistant to tetracycline, chloramphenicol, sulfonamide, and ampicillin, respectively.

Sulfonamide was the second most common antimicrobial with resistance among the *E. coli* isolates in this study. In 2012, in the Federal District region, 74.8% and 70.1% strains in pig feces on breeding farms were resistant to sulfonamides and tetracyclines, respectively [55]. Unlike these reports [56,57,58,59,60], tetracycline resistance frequencies in this study were low, with 28 doxycycline- and tetracycline-resistant isolates each (53.9%).

Forty-four (84.61%) isolates presented resistance to chloramphenicol, which has been banned from use in animals in Brazil since 2003 [61]. The results obtained were corroborated by Mangal et al. [51], who verified resistance to chloramphenicol in 30 isolates (73.2%) in India. Drummond and Perecmanis [52] also identified 34.7% chloramphenicol-resistant strains in pig feces in the Federal District. The persistence of this resistance can be attributed to the maintenance of chloramphenicol resistance genes through co-selection with other resistance and virulence genes [55,56,62,63,64]. However, further studies are necessary to confirm this.

Resistance to the antimicrobials streptomycin and sulphonamide was 80.8% and 84.6%, respectively, which were different from the results of Mangal et al. [51], who obtained 100% sensitivity to streptomycin and sulphonamide in retail pork in India. Streptomycin resistance is a common finding in *E. coli* isolated from animals and animal products and, along with ampicillin resistance, is among the resistance phenotypes that are frequently co-transferred with sulphonamide resistance [65], which may explain the results of this study since the isolates also showed 81.1% resistance to sulphonamides.

The lowest resistance frequencies were observed for first-generation cephalosporin cefazolin (9.6%) and third-generation ceftazidime (7.7%), followed by gentamicin (13.5%). Moennighoff et al. [66] identified less than 15% resistance to gentamicin in pigs in Germany, as in this study. Third-generation cephalosporins are part of the so-called highest priority antimicrobials, which are critically important in human medicine, together with quinolones, macrolides, ketolides, and glycopeptides [67]. In this study, 7.7% *E. coli* isolates were ceftazidime-resistant, being the first report of this resistance in pig carcasses in the region (Federal District and Central West).

All 52 isolates were resistant to at least two antimicrobials. The presence of resistance to seven antimicrobials was detected in 15 isolates, which is the highest frequency of multidrug resistance in this study. However, three (5.77%) isolates were resistant to 10 antimicrobials. Only two isolates were sensitive to 12 antimicrobials out of the 13 tested, with the most sensitive antimicrobials being ceftazidime (90.4%) and gentamicin (86.5%).

Antimicrobial resistance is a global problem, posing a growing threat to public health [55]. Resistant bacteria can be transmitted to humans when consuming and handling contaminated food through direct contact with animals and the distribution of these bacteria in the environment [56]. The results obtained in this study demonstrate phenotypic resistance in slaughterhouses A and B, and most *E. coli* isolates showed multidrug resistance, which may represent an important source of dissemination of antimicrobial resistance in the environment.

### 3.5. Eae, stx1, and stx2 Virulence Gene Detection in E. coli

**Figure 6:**
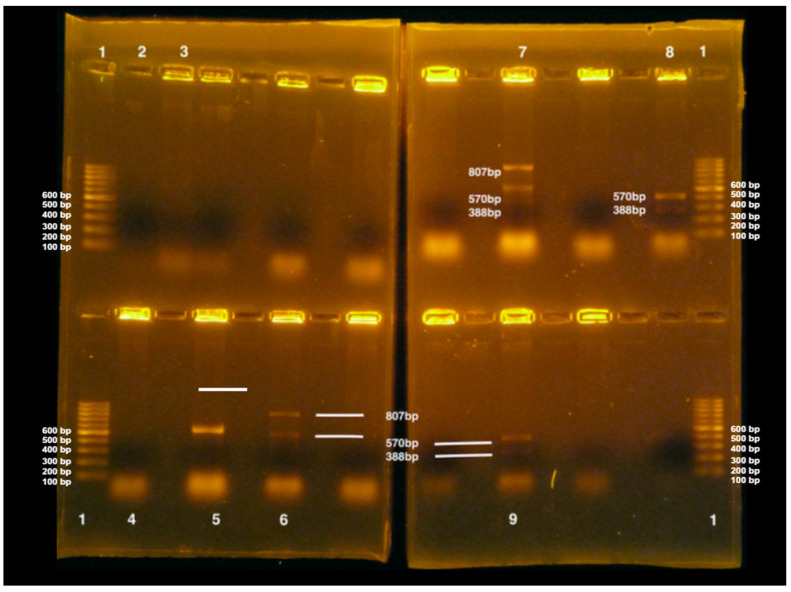
Detection of virulence genes *stx1*, *stx2,* and *eae* by multiple PCR in *E. coli* isolates in pig carcasses from slaughterhouse B in two gels. (1) 100 bp marker, (2) negative control, (3) positive control for *stx1* gene (388 bp) in *E. coli*, (4) isolate 10D—negative, (5) isolate 10E—positive for *eae* gene (570 bp), (6) isolate 11A—positive for *eae* (570 bp) and *stx2* (807 bp) genes, (7) isolate 11D—positive for *eae* (570 bp), *stx1* (388 bp) and *stx2* (807 bp) genes, (8) isolate 12A—positive for *eae* genes (570 bp) and *stx1* (388 bp), (9) isolate 12D—positive for *eae* (570 bp) and *stx1* (388 bp) genes.

The *eae, stx1*, and *stx2* genes were identified by PCR in 24/52 (46.15%), 16/52 (30.8%), and 9/52 (17.3%) *E. coli* isolates, respectively. Only three (5.8%) isolates (5D, 8A, and 11D) concomitantly presented all three genes (Figure 6).

*E. coli* with *stx1* and *stx2* genes can be called STEC [57,68]. Among the isolates, 11 isolates had *stx1*, four isolates had *stx2*, and five isolates contained both *stx1* and *stx2* genes. These genes are the main markers for STEC detection using PCR [58]. Corroborating the results regarding the detection of these genes in this study, Bouvet et al. [59] detected 48 STEC isolates in pig carcasses in slaughterhouses in France, 47 of which had the *stx2* gene, and one isolate had the *stx1* gene. However, this study identified more isolates containing the *stx1* gene than *stx2*. These authors also studied the *eae* gene but did not identify it, considering the isolates to be of low potential danger to public health, as the simple finding of *stx* genes in food would not pose a threat to human consumption [60].

In contrast, in this study, 24 isolates containing the *eae* gene were identified; of these, five also harbored the *stx1* gene (1E, 4A, 6B, 12A, and 12D), and three harbored the *stx2* gene (3E, 9A, and 11A). Furthermore, three isolates presented all three genes (*stx1*, *stx2*, and *eae*) that could represent a risk to public health, as they contained one of the enhanced factors of STEC infection capacity, the intimin gene [62,63].

In Brazil, STEC outbreaks related to pork consumption have not been described to date. However, three outbreaks between 2014 and 2018 related to STEC O157:H7 have been described in Canada, with the detection of the *stx2a* and *stx1a + stx2a* genes and *eae* in the isolates [64], corroborating the importance of these findings, though the isolates were not serotyped.

In Slaughterhouse A, the *stx1* gene was identified in isolates from four points during the slaughter process: after bleeding (1E: jowl), before evisceration (4A: shank, 4D: shoulder), after evisceration (5D: abdomen), and after washing the carcass prior to cooling (6B: loin). The *stx2* gene was identified after manual skinning (3E: jowl) and evisceration (5D: shoulder). The *eae* gene was identified at all slaughter stages: after bleeding (1B: loin, 1C: shoulder, and 1E: jowl), after epilation (2A: shank), after manual skin (3A: shank, 3B: loin, 3E: jowl), before evisceration (4A: shank, 4D: abdomen), after evisceration (5A: shank, 5D: abdomen), and after washing the carcass prior to cooling (6B: loin, 6D: abdomen).

The results of the detection of virulence genes, together with the antibiograms of the isolates in this study, are presented in Table 11.

In slaughterhouse B, the *stx1* gene was identified in *E. coli* isolates from all carcass points after bleeding (7A, 7B, 7C, 7D, 7E), shoulder before evisceration (10C), abdomen after evisceration (11D), and loin and abdomen after washing the carcass prior to cooling (12B, 12D). The *stx2* gene was identified in the shank after bleeding (7A), shank and loin after epilation (8A, 8B), jowl and shoulder after manual skin process (9A, 9C), and shank and abdomen after evisceration (11A and 11D). The *eae* gene was not identified after bleeding but was identified in the shank after depilation (8A), shank and loin after manual skin (9A, 9B), shank and jowl before evisceration (10A, 10E), shank and abdomen after evisceration (11A, 11D), and shank, loin, and abdomen after washing the carcass prior to cooling (12A, 12B, and 12D).

Therefore, the transmission of STEC can potentially occur at all pig slaughter stages in the Federal District region.

### 3.6. In Vitro Biofilm Formation Capacity Evaluation

The in vitro biofilm-forming capacities of 51 *E. coli* isolates were tested. Of these, 22 isolates were from Slaughterhouse A, and 29 were from Slaughterhouse B. The ability to form biofilms with respect to the incubation time was not significantly different (*p* < 0.086).

Regarding temperature variation at 10 °C, the ability to not form biofilm was greater compared to that at other temperature ranges, while the ability to form moderate and strong biofilm was more frequent at 37 and 24 °C.

Regarding the collection stage during the slaughterhouse process, there was a statistically significant difference (*p* < 0.0001): the toilet had a higher frequency of non-biofilm-forming *E. coli*, while the post-evisceration stage had a higher frequency of strong biofilm-forming *E. coli*.

At the isolated points of the carcass, *E. coli* also showed a significant difference (*p* < 0.0001), with the isolates from the jowl region showing a higher frequency of non-biofilm-forming *E. coli*, whereas the isolates from the shank region showed a higher frequency of strong biofilm-forming *E. coli*.

Twenty *E. coli* isolates with characteristic virulence factors for STEC (*stx1* and *stx2* genes) were evaluated for their biofilm-forming capacity. Of these isolates, eight showed weak or no formation capacity at the different temperatures evaluated (1E, 4D, 6B, 7E, 8A, 9A, 10C, and 12D). Four isolates showed a moderate or strong capacity for biofilm formation at the three temperatures evaluated (4A, 7A, 7 B, and 11A). Another eight isolates (3E, 5D, 7C, 7D, 8 B, 9C, 11D, and 12A) showed varying biofilm-formation capabilities according to the temperatures studied.

Of the 11 isolates with the *stx1* gene, two (1E and 6B) did not show biofilm formation capacity, two (10C and 12D) showed weak biofilm formation, and only one (4A) showed strong biofilm formation capacity at the three tested temperatures. The other six isolates had biofilm-forming capacities that varied with temperature.

Two *E. coli* isolates with the *stx2* gene presented weak biofilm formation capacity at 37 °C (3E and 9A), and two presented moderate biofilm formation at 24 °C (8B and 9A). Two isolates had moderate capacity at 37 °C (8B and 11A) and 24 °C (3E and 11A) each. No isolate with the *stx2* gene was non-forming at the three temperatures and strongly forming at temperatures of 37 and 24 °C.

Evaluation of the joint presence of the *stx1* and *stx2* virulence genes in an isolate and its biofilm-forming capacity revealed that isolate 8A was a weak former and 7A was a strong former at the three temperatures tested. Isolates 5D and 11D were weak formers at 10 °C and isolate 9C at 37 °C. At 24 °C, isolates 9C and 11D showed moderate biofilm-forming capacities. At 37 °C, isolates 5D and 11D showed a moderate capacity for biofilm formation, and 11D also showed it at 24 °C. Isolates 5D and 9C showed a strong ability to form biofilm at 24 and 10 °C, respectively. Table 12 presents the results.

In contrast to the results of this study, Milan and Timm [69] detected STEC isolates of bovine origin in Brazil that were incapable of forming biofilms.

Evaluating the isolates with virulence genes according to their biofilm formation capacity and antimicrobial resistance infer that isolates 4A, 7A, 7B, and 11A have disease-causing factors, can form moderate and strong biofilms at different temperatures, and are resistant to multiple antimicrobials. Isolate 5D also has multidrug resistance, with moderate and strong biofilm formation capacity at 37 and 24 °C, respectively. Therefore, these isolates are relevant to public health. Corroborating these results, Sanchez et al. [70] evaluated isolates from human samples and detected multidrug-resistant *E. coli* isolates capable of forming biofilms.

Therefore, the results presented in this study on STEC virulence genes in swine carcasses in the Federal District are relevant because of the presence of isolates containing the *stx1* and *stx2* genes as well as antimicrobial multiresistance phenotypes.

This is the first study in Brazil to evaluate the capacity to form biofilms, the presence of *stx1*, *stx2,* and *eae* virulence genes, and antimicrobial resistance in *E. coli* isolates from different locations on the pig carcass in the process stages of slaughter. The results demonstrate varied biofilm formation capabilities and the presence of STEC isolates at different slaughter stages in different locations on carcasses, thus making them relevant to public health.

## 4. Conclusions

In this study, the *Enterobacteriaceae* and *E. coli* counts in pig carcasses were measured, which were relevant as quality controls between slaughter processing stages as they revealed the stages with the greatest contamination.

Phenotypic characterization using disk diffusion technique demonstrated high antimicrobial multidrug resistance in *E. coli* isolates. Furthermore, they were highly sensitive to third-generation cephalosporins and ceftazidime, which are considered critically important for treating serious bacterial infections in humans. However, these isolates are already resistant to quinolones and macrolides, such as nalidixic acid and erythromycin, which are also on the World Health Organization (WHO) list; therefore, there is a warning about antimicrobial use in animals.

In *E. coli* isolates, virulence genes for STEC have been isolated; these are *stx1* and *stx2* genes, in large numbers, with or without the *eae* intimin gene, which can enhance the virulence of the strains. Another factor identified, which may enhance virulence, in addition to keeping such genes persistent and disseminating them along with multidrug resistance in the environment, is the ability to form biofilms, which varies at different temperatures, with few non-biofilm formers.

The presence of these pathogens, together with the indicator microorganism counts, reflect the hygienic conditions of the slaughterhouses and technological processes of slaughter in the establishments evaluated; they can contribute to the industry in general as this is the first study on the counts of these microorganisms on the pig carcass surface and other parts during the different stages of slaughter in Brazil.

This is the first microbial characterization study on pig carcasses in the Federal District, both in different parts of the carcass and at different stages of slaughter, demonstrating the presence of *stx1*, *stx2*, and *eae* genes, multidrug resistance, and various biofilm formation in *E. coli*. Both pathogen and virulence gene identification as well as antimicrobial resistance can represent a risk to public health, given their presence at different slaughter stages.

## Figures and Tables

**Table 1:** List of primers used to detect virulence genes in isolates of *E. coli*.

Gene	Sequence of Nucleotides (5′-3′)	Size (pb)	Annealing Temperature (°C)	Reference
*Stx-1*	F-AGA GCG ATG TTA CGG TTT G R-TTG CCC CCA GAG TGG ATG	388	50	[35]
*Stx-2*	F-TGG GTT TTT CTT CGG TAT C R-GAC ATT CTG GTT GAC TCT CTT	807	50	[35]
*eae*	F-AGG CTT CGT CAC AGT TG R-CCA TCG TCA CCA GAG GA	570	50	[35]

**Table 2:** *Enterobacteriaceae* and *E. coli* count results in UFC/cm^2^ at different parts/points of the carcass, after bleeding stage, during the technological processing of pig slaughter in slaughterhouses A and B located in the Federal District region. The average counts of all parts of the carcass were converted into log UFC/cm^2^. (US* Unviable/lost sample; NG** No growth).

Slaughter Technological Processing Stage	Visits (Slaughterhouse A and B)	Microorganism	Carcass Part/Point (Count in CFU/cm^2^)					Average Carcass Count (log CFU/cm^2^)
			1 Shank	2 Loin	3 Shoulder	4 Abdomen	5 Jowl	
After bleeding	1st visit (slaughterhouse A)	*Enterobacteriaceae*	2.5 × 10^2^ 2.3log	1.3 × 10^1^ 1.1log	9.2 × 10^2^ 2.9log	2.6 × 10^2^ 2.4log	US* 0	2.55
		*E. coli*	1.0 × 10^2^ 2log	0.2 × 10^1^ 0.3log	7.0 × 10^1^ 1.84log	2.1 × 10^1^ 1.3log	US* 0	1.68
	1st visit (slaughterhouse B)	*Enterobacteriaceae*	8.0 × 10^1^ 1.9log	6.1 × 10^1^ 1.7log	4.0 × 10^1^ 1.6log	6.0 × 10^1^ 1.7log	1.4 × 10^2^ 2.1log	1.88
		*E. coli*	0.4 × 10^1^ 0.6log	0.3 × 10^1^ 0.4log	10^4^ 4log	10^4^ 4log	10^4^ 4log	3.77
	2nd visit (slaughterhouse B)	*Enterobacteriaceae*	NG** 0	0.8 × 10^1^ 0.9log	1.2 × 10^3^ 3.07log	0.6 × 10^2^ 1.7log	9.8 × 10^2^ 2.9log	2.65
		*E. coli*	NG** 0	NG** 0	2.8 × 10^2^ 2.4log	NG** 0	2.1 × 10^2^ 2.3log	1.99

**Table 3:** *Enterobacteriaceae* and *E. coli* count results in UFC/cm^2^ at different parts/points of the carcass after the epilation process during the technological processing of pig slaughter in slaughterhouses A and B located in the Federal District region. The average counts of all parts of the carcass were converted into log UFC/cm^2^. (NG** No growth).

Slaughter Technological Processing Stage	Visits (Slaughterhouse A and B)	Microorganism	Carcass Part/Point (Count in CFU/cm^2^)					Average Carcass Count (log CFU/cm^2^)
			1 Shank	2 Loin	3 Shoulder	4 Abdomen	5 Jowl	
After epilation process	1st visit (slaughterhouse A)	*Enterobacteriaceae*	3.9 × 10^1^ 1.5log	3.2 × 10^2^ 2.5log	3.8 × 10^1^ 1.5log	2.1 × 10^2^ 2.3log	2.8 × 10^2^ 2.4log	2.24
		*E. coli*	2.1 × 10^1^ 1.3log	1.3 × 10^1^ 1.1log	0.7 × 10^1^ 0.8log	0.2 × 10^1^ 0.3log	0.4 × 10^1^ 0.6log	0.97
	1st visit (slaughterhouse B)	*Enterobacteriaceae*	0.4 × 10^1^ 0.6log	2.9 × 10^1^ 1.4log	4.9 × 10^1^ 1.6log	2.0 × 10^1^ 1.3log	3.3 × 10^1^ 1.5log	1.43
		*E. coli*	1.3 × 10^1^ 1.1log	1.2 × 10^1^ 1log	1.9 × 10^1^ 1.2log	0.9 × 10^1^ 0.9log	2.1 × 10^1^	1.17
	2nd visit (slaughterhouse B)	*Enterobacteriaceae*	1.2 × 10^2^ 2log	6.0 × 10^1^ 1.7log	2.8 × 10^2^ 2.4log	3.4 × 10^2^ 2.5log	4.8 × 10^2^ 2.6log	2.40
		*E. coli*	NG**	NG**	NG**	NG**	NG**	0

**Table 4:** *Enterobacteriaceae* and *E. coli* count results in UFC/cm^2^ at different parts/points of the carcass, after manual skin process during the technological processing of pig slaughter, in slaughterhouses A and B located in the Federal District region. The average counts of all parts of the carcass were converted into log UFC/cm^2^. (US* Unviable/lost sample; NG** No growth).

Slaughter Technological Processing Stage	Visits (Slaughterhouse A and B)	Microorganism	Carcass Part/Point (Count in CFU/cm^2^)					Average Carcass Count (log CFU/cm^2^)
			1 Shank	2 Loin	3 Shoulder	4 Abdomen	5 Jowl	
After manual skin process	1st visit (slaughterhouse A)	*Enterobacteriaceae*	3.5 × 10^2^ 2.5log	US* 0	3.2 × 10^1^ 1.5log	4.2 × 10^2^ 2.6log	3.0 × 10^2^ 2.4log	2.4
		*E. coli*	9.8 × 10^1^ 1.9log	1.0 × 10^1^ 1log	0.9 × 10^1^ 0.9log	1.3 × 10^1^ 1.1log	US* 0	1.51
	1st visit (slaughterhouse B)	*Enterobacteriaceae*	7.7 × 10^1^ 1.8log	4.0 × 10^1^ 1.6log	2.8 × 10^1^ 1.4log	0.4 × 10^1^ 0.6log	2.9 × 10^2^ 2.4log	1.94
		*E. coli*	2.9 × 10^1^ 1.4log	3.0 × 10^1^ 1.4log	1.5 × 10^1^ 1.1log	1.1 × 10^1^ 1log	10^4^ 4log	3.30
	2nd visit (slaughterhouse B)	*Enterobacteriaceae*	2.4 × 10^2^ 2.3log	1.2 × 10^2^ 2log	NG**	0.5 × 10^1^ 0.6log	NG**	1.86
		*E. coli*	NG**	NG**	2.0 × 10^1^ 1.3log	NG**	NG**	0.6

**Table 5:** *Enterobacteriaceae* and *E. coli* count results in UFC/cm^2^ at different parts/points of the carcass before the evisceration stage during the technological processing of pig slaughter in slaughterhouses A and B located in the Federal District region. The average counts of all parts of the carcass were converted into log UFC/cm^2^. (NG** No growth).

Slaughter Technological Processing Stage	Visits (Slaughterhouse A and B)	Microorganism	Carcass Part/Point (Count in CFU/cm^2^)					Average Carcass Count (log CFU/cm^2^)
			1 Shank	2 Loin	3 Shoulder	4 Abdomen	5 Jowl	
Before evisceration	1st visit (slaughterhouse A)	*Enterobacteriaceae*	4.3 × 10^3^ 2.6log	2.3 × 10^2^ 2.3log	2.5 × 10^2^ 2.3log	5.0 × 10^1^ 1.6log	2.7 × 10^2^ 2.4log	2.39
		*E. coli*	1.7 × 10^2^ 2.2log	2.4 × 10^1^ 1.3log	NG**	2.7 × 10^1^ 1.4log	0.9 × 10^1^ 0.9log	1.66
	1st visit (slaughterhouse B)	*Enterobacteriaceae*	2.7 × 10^1^ 31.4log	3.9 × 10^1^ 1.5log	3.6 × 10^1^ 1.5log	0.7 × 10^1^ 0.8log	6.6 × 10^1^ 1.8log	1.54
		*E. coli*	1.1 × 10^1^ 1log	1.3 × 10^1^ 1.1log	1.4 × 10^1^ 1.1log	0.7 × 10^1^ 0.8log	0.8 × 10^1^ 0.9log	1.02
	2nd visit (slaughterhouse B)	*Enterobacteriaceae*	0.6 × 10^1^ 1.7log	2.0 × 10^1^ 1.3log	0.1 × 10^1^ 0	NG**	NG**	1.2
		*E. coli*	2.0 × 10^1^ 1.3log	NG**	NG**	NG**	NG**	0.6

**Table 6:** *Enterobacteriaceae* and *E. coli* count results in UFC/cm^2^ at different parts/points of the carcass after the evisceration stage during the technological processing of pig slaughter in slaughterhouses A and B located in the Federal District region. The average counts of all parts of the carcass were converted into log UFC/cm^2^. (US* Unviable/lost sample; NG** No growth).

Slaughter Technological Processing Stage	Visits (Slaughterhouse A and B)	Microorganism	Carcass Part/Point (Count in CFU/cm^2^)					Average Carcass Count (log CFU/cm^2^)
			1 Shank	2 Loin	3 Shoulder	4 Abdomen	5 Jowl	
After evisceration	1st visit (slaughterhouse A)	*Enterobacteriaceae*	6.2 × 10^1^ 1.7log	2.8 × 10^1^ 1.4log	4.4 × 10^2^ 2.6log	5.4 × 10^1^ 1.7log	2.2 × 10^2^ 2.3log	2.20
		*E. coli*	3.4 × 10^2^ 2.5log	3.8 × 10^1^ 1.5log	2.9 × 10^1^ 1.4log	US*	2.9 × 10^1^ 1.4log	2.03
	1st visit (slaughterhouse B)	*Enterobacteriaceae*	1.0 × 10^3^ 3log	1.7 × 10^2^ 2.2log	3.1 × 10^2^ 2.4log	1.3 × 10^3^ 3.1log	1.3 × 10^3^ 3.1log	2.91
		*E. coli*	10^6^ 6log	10^6^ 6log	10^6^ 6log	10^6^ 6log	10^6^ 6log	6.0
	2nd visit (slaughterhouse B)	*Enterobacteriaceae*	1.2 × 10^2^ 2log	8.0 × 10^2^ 2.9log	3.6 × 10^1^ 1.5log	1.4 × 10^2^ 2.1log	0.4 × 10^1^ 0.6log	2.3
		*E. coli*	2.0 × 10^1^ 1.3log	6.0 × 10^1^ 1.7log	NG**	NG**	NG**	1.20

**Table 7:** *Enterobacteriaceae* and *E. coli* count results in UFC/cm^2^ at different parts/points of the carcass after washing the carcass and before moving them into the cooling chamber of pig slaughterhouses A and B located in the Federal District region. The average counts of all parts of the carcass were converted into log UFC/cm^2^. (NG** No growth).

Slaughter Technological Processing Stage	Visits (Slaughterhouse A and B)	Microorganism	Carcass Part/Point (Count in CFU/cm^2^)					Average Carcass Count (log CFU/cm^2^)
			1 Shank	2 Loin	3 Shoulder	4 Abdomen	5 Jowl	
After wash and before enter the cooling chamber	1st visit (slaughterhouse A)	*Enterobacteriaceae*	1.1 × 10^2^ 2log	5.1 × 10^1^ 1.6log	3.3 × 10^1^ 1.5log	3.2 × 10^1^ 1.5log	1.8 × 10^1^ 1.2log	1.68
		*E. coli*	3.3 × 10^1^ 1.5log	8.7 × 10^1^ 1.9log	4.4 × 10^1^ 1.6log	0.2 × 10^1^ 0.3log	4.0 × 10^1^ 1.6log	1.61
	1st visit (slaughterhouse B)	*Enterobacteriaceae*	7.5 × 10^1^ 1.8log	7.2 × 10^1^ 1.8log	1.0 × 10^2^ 2log	1.5 × 10^2^ 2.1log	1.5 × 10^2^ 2.1log	2.03
		*E. coli*	10^4^ 4log	10^4^ 4log	10^4^ 4log	10^4^ 4log	10^4^ 4log	4.0
	2nd visit (slaughterhouse B)	*Enterobacteriaceae*	NG** 0	NG** 0	NG** 0	NG** 0	NG** 0	0
		*E. coli*	NG** 0	NG** 0	NG** 0	NG** 0	NG** 0	0

**Table 8:** Identification of *E. coli* isolates by stage of the technological processing of slaughter and location on the carcass, in slaughterhouses A in 1st visit and B in 2nd visit.

Slaughtering Stage	Slaughterhouse/Visit	Shank	Loin	Shoulder	Abdomen	Jowl
After bleeding	A (1st visit)	1A	1B	1C	1D	1E
	B (2nd visit)	7A	7B	7C	7D	7E
After epilation	A (1st visit)	2A	2B	2C	ND	ND
	B (2nd visit)	8A	8B	8C	8D	8E
After manual skin	A (1st visit)	3A	3B	ND	3D	3E
	B (2nd visit)	9A	9B	9C	9D	9E
Before evisceration	A (1st visit)	4A	ND	ND	4D	4E
	B (2nd visit)	10A	10B	10C	10D	10E
After evisceration	A (1st visit)	5A	5B	5C	5D	ND
	B (2nd visit)	11A	11B	11C	11D	11E
After final wash	A (1st visit)	6A	6B	ND	6D	6E
	B (2nd visit)	12A	12B	ND	12D	12E

ND = not detected.

**Table 9:** Antibiogram of 52 *E. coli* isolates from slaughterhouses A and B located in the Federal District region, using the disk-diffusion method and interpreted, according to CLSI parameters [34], as resistant I, intermediate (I), or sensitive (S).

Antimicrobial	Resistant	Intermedite	Sensitive
**Nalidixic acid**	1A-1B-1C-1D; 2A-2B-2C; 3A-3D; 4A-4E; 5A-5B-5C-5D; 6A-6B-6D-6E; 7B-7C-7D; 8B-8C-8D; 9A-9B-9D-9E; 10A-10C-10D; 11A-11B-11C-11D-11E; 12E	3B-3E 7A-7E 8A	1E; 4D 8E; 9C ‘10B-10E 12A-1-B-12D
**Amoxicillin**	1A-1B-1C-1D-1E; 2A-2B-2C 3A-3B-3D-3E; 4A-4D-4E; 5A-5B-5C-5D; 6A-6B-6E; 7A-7B-7D-7E; 8A-8B-8C-8D-8E; 9A-9B-9D 10A, 10B, 10D 11A-11B-11C-11D-11E 12A-12B-12D-12E	-	6D 7C 9-C-9E 1-C-10E
**Ampicillin**	1A-1B-1C-1D-1E; 2A-2B-2C 3A-3B-3D-3E; 4A-4D-4E - 5A-5B-5C-5D; 6A-6B-6E 7A-7B-7D-7E 8A-8B-8C-8D-8E; 9A-9B-9D; 10A, 10B, 10D 11A-11B-11C-11D-11E 12A-12B-12D-12E	-	6D 7C 9-C-9E 1-C-10E
**Cefazolin**	1D 8A-8D 10A-10C	1A-1B-1C-1E; 2A-2B-2C; 3A-3B-3D-3E; 4A-4D-4E; 5A-5B-5C-5D; 6A-6B-6D-6E 8B-8C-8E; 9A; 11E	7A-7B-7C-7D-7E; 9B-9C-9D-9E; 10B-10D-10E; 11A-11B-11C-11D 12A-12B-12D-12E
**Ceftazidime**	2A-2B 8A 9C	1C	1A-1B-1D-1E; 2C; 3A-3B-3D-3E; 4A-4D-4E; 5A-5B-5C-5D; 6A-6B-6D-6E 7A-7B-7C-7D-7E 8B-8C-8D-8E; 9A-9B-9D-9E; 10A-10B-10C-10D-10E 11A-11B-11C-11D-11E; 12A-12B-12D-12E
**Ciprofloxacin**	1C-1D; 2C; 3B-3D-3E; 4E 5D; 6D 7C; 9A 10A-10D	1A; 4A; 5B; 6A 7A-7E; 8A 9D; 10C	1B-1E; 2A-2B; 3A 4D 5A-5C; 6B-6E 7B-7D; 8B-8C-8D-8E 9B-9C-9E; 10B-10E 11A-11B-11C-11D-11E 12A-12B-12D-12E
**Chloramphenicol**	1A-1C-1D; 2A-2B-2C 3A-3B-3D-3E; 4A-4E 5A-5B-5D; 6A-6B-6D-6E 7A-7B-7C-7D-7E; 8A-8B-8C-8E; 9A-9B-9D-9E 10A-10B-10C-10D-10E 11A-11B-11C-11D-11E; 12E	8D	1B-1E 4D; 5C 9C 12A-12B-12D
**Doxycycline**	1A-1B; 6D 7A-7B-7C-7D-7E 8A-8B-8C-8D-8E 9A-9B-9D-9E 10A-10B-10C-10D-10E 11A-11B-11C-11D-11E; 12E	1D-1E	1C; 2A-2B-2C 3A-3B-3D-3E; 4A-4D-4E 5A-5B-5C-5D 6A-6B-6E 9C 12A-12B-12D
**Streptomycin**	1A-1C-1D-1E; 2A-2B-2C; 3A-3B-3D-3E; 4A-4D-4E; 5A-5C-5D; 6A-6B-6D-6E 7A-7B-7C-7D-7E 8A-8B-8C-8E; 9A-9B-9D; 10A-10B-10E 11A-11B-11C-11D-11E; 12E	8D 9E 10C-10D	1B 5B 9C 12A-12B-12D
**Erythromycin**	1A-1B-1C-1D-1E; 2A-2B-2C 3A-3B-3D-3E; 4A-4D-4E 5A-5B-5C-5D; 6A-6B-6D-6E 7A; 8A-8B-8C-8D 10C-10D-10E 11A-11B-11C-11D-11E; 12E	-	7B-7C-7D-7E; 8E 9A-9B-9C-9D-9E 10A-10B 12A-12B-12D
**Gentamicin**	1C-1D 2C 4E; 5B-5D 7C	-	1A-1B-1E; 2A-2B; 3A-3B-3D-3E; 4A-4D; 5A-5C; 6A-6B-6D-6E 7A-7B-7D-7E; 8A-8B-8C-8D-8E; 9A-9B-9C-9D-9E 10A-10B-10C-10D-10E 11A-11B-11C-11D-11E 12A-12B-12D-12E
**Sulfonamide**	1A-1C-1D-1E; 2A-2B-2C 3A-3B-3D-3E; 4A-4D-4E 5A-5B-5C-5D; 6A-6B-6D-6E 7A-7B-7C-7D-7E; 8C-8E; 9A-9B-9E; 10A-10B-10C-10D-10E 11A-11B-11C-11D-11E 12D-12E	-	1B 8A-8B-8D 9C-9D 12A-12B
**Tetracycline**	1A-1B; 6D 7A-7B-7C-7D-7E 8A-8B-8C-8D-8E 9A-9B-9D-9E 10A-10B-10C-10D-10E 11A-11B-11C-11D-11E; 12E	-	1C-1D-1E; 2A-2B-2C 3A-3B-3D-3E; 4A-4D-4E 5A-5B-5C-5D; 6A-6B-6E 9C 12A-12B-12D

**Table 10:** Frequency results of 52 *E. coli* isolate antibiograms from slaughterhouses A and B located in the Federal District region, using the disk-diffusion method and interpreted, according to CLSI parameters [34], as resistant I, intermediate (I), or sensitive (S).

Antimicrobial	Number of Resistant Isolates (%)	Number of Intermediate Resistance Isolates (%)	Number of Sensitive Isolates (%)	Total of Resistant and Intermediate Isolates (%)
Nalidixic acid (NAL)	73% (38/52)	9.6% (5/52)	17.4% (9/52)	82.6% (43/52)
Amoxicillin (AMO)	88.4% (46/52)	0% (0/0)	11.6% (6/52)	88.4% (46/52)
Ampicillin (AMP)	88.4% (46/52)	0% (0/0)	11.6% (6/52)	88.4% (46/52)
Cefazolin (CFZ)	9.6% (5/52)	51.9% (27/52)	38.5% (20/52)	61.5% (32/52)
Ceftazidime (CAZ)	7.6% (4/52)	2% (1/52)	90.4% (47/52)	9.6% (5/52)
Ciprofloxacin (CIP)	25% (13/52)	17.3% (9/52)	57.7% (30/52)	42.3% (22/52)
Chloramphenicol (CLO)	82.6% (43/52)	2% (1/52)	15.4% (8/52)	84.6% (44/52)
Doxycycline (DOX)	53.7% (28/52)	4% (2/52)	42.3% (22/52)	57.7% (30/52)
Streptomycin (EST)	80.7% (42/52)	7.7% (4/52)	11.6% (6/52)	88.4% (46/52)
Erythromycin (ERI)	71.1% (37/52)	0% (0/0)	28.9% (15/52)	71.1% (37/52)
Gentamicin (GEN)	13.5% (7/52)	0% (0/0)	86.5% (45/52)	13.5% (7/52)
Tetracycline (TET)	53.7% (28/52)	0% (0/0)	46.3% (24/52)	53.7% (28/52)
Sulfonamide (SUL)	84.6% (44/52)	0% (0/0)	15.4% (8/52)	84.6% (44/52)

**Table 11:** Antibiogram, PCR detection of the virulence genes *stx1*, *stx2,* and *eae* in *E. coli* isolates from pig carcasses from slaughterhouses A and B located in the Federal District.

Isolates	Slaughterhouse	Antimicrobial Resistance	Virulence Gene
**1B**	A	AMO AMP ERI DOX TET NAL	*eae*
**1C**	A	AMO AMP ERI EST GEN SUL CLO NAL CIP	*eae*
**1E**	A	AMO AMP ERI EST SUL	*eae*, *stx1*
**2A**	A	AMO AMP CAZ ERI EST SUL CLO NAL	*eae*
**3A**	A	AMO AMP EST ERI SUL CLO NAL	*eae*
**3B**	A	AMO AMP ERI EST SUL CLO CIP	*eae*
**3E**	A	AMO AMP ERI EST SUL CLO CIP	*eae*, *stx2*
**4A**	A	AMO AMP ERI EST SUL CLO NAL	*eae*, *stx1*
**4D**	A	AMO AMP ERI EST SUL	*eae*, *stx1*
**5A**	A	AMO AMP ERI EST SUL CLO NAL	*eae*
**5D**	A	AMO AMP ERI EST GEN SUL CLO NAL CIP	*eae*, *stx1*, *stx2*
**6B**	A	AMO AMP ERI EST SUL CLO NAL	*eae*, *stx1*
**6D**	A	ERI EST SUL DOX TET CLO NAL CIP	*eae*
**7A**	B	AMO AMP ERI EST SUL DOX TET CLO	*stx1*, *stx2*
**7B**	B	AMO AMP EST SUL DOX TET CLO NAL	*stx1*
**7C**	B	EST GEN SUL DOX TET CLO NAL CIP	*stx1*
**7D**	B	AMO AMP EST SUL DOX TET CLO NAL	*stx1*
**7E**	B	AMO AMP EST SUL DOX TET CLO	*stx1*
**8A**	B	AMO AMP CFZ CAZ ERI EST DOX TET CLO	*eae*, *stx1*, *stx2*
**8B**	B	AMO AMP ERI EST DOX TET CLO NAL	*stx2*
**8E**	B	AMO AMP EST SUL DOX TET CLO	*eae*
**9A**	B	AMO AMP EST SUL DOX TET CLO NAL CIP	*eae*, *stx2*
**9B**	B	AMO AMP EST SUL DOX TET CLO NAL	*eae*
**9C**	B	CAZ	*stx1*, *stx2*
**10B**	B	AMO AMP EST SUL DOX TET CLO	*eae*
**10C**	B	CFZ ERI SUL DOX TET CLO NAL	*stx1*
**10E**	B	EST SUL DOX TET CLO	*eae*
**11A**	B	AMO AMP EST SUL DOX TET CLO NAL	*eae*, *stx2*
**11D**	B	AMO AMP EST SUL DOX TET CLO NAL	*eae*, *stx1*, *stx2*
**12A**	B	AMO AMP ERI	*eae*, *stx1*
**12B**	B	AMO AMP	*eae*
**12D**	B	AMO AMP ERI SUL	*eae*, *stx1*

AMO = amoxicillin, AMP = ampicillin, CFZ = cefazolin, CAZ = ceftazidime, ERI = erythromycin, EST = streptomycin, GEN = entamicinacin, SUL = Sulfonamide, DOX = doxycycline, TET = tetracycline, CLO = chloramphenicol, NAL = nalidixic acid, CIP = ciprofloxacin.

**Table 12:** Antimicrobial resistance and biofilm formation capacity of *E. coli* containing the *stx1* and *stx2* virulence genes, at different temperatures, from pig carcasses from slaughterhouses A and B.

Isolates	Virulence Genes	Biofilm Formation Capacity			Antimicrobial Resistance
		37 °C	24 °C	10 °C	
1E	*stx1*	NF	NF	NF	AMO-AMP-ERI-EST-SUL
3E	*stx2*	WF	MF	WF	AMO-AMP-ERI-EST-SUL-CLO-CIP
4A	*stx1*	SF	SF	SF	AMO-AMP-ERI-EST-SUL-CLO-NAL
4D	*stx1*	WF	WF	NF	AMO-AMP-ERI-EST-SUL
5D	*stx1*, *stx2*	MF	FFO	WF	AMO-AMP-ERI-EST-GEN-SUL-CLO-NAL-CIP
6B	*stx1*	NF	NF	NF	AMO-AMP-ERI-EST-SUL-CLO-NAL
7A	*stx1*, *stx2*	SF	SF	SF	AMO-AMP-ERI-EST-SUL-DOX-TET-CLO
7B	*stx1*	MF	SF	SF	AMO-AMP-EST-SUL-DOX-TET-CLO-NAL
7C	*stx1*	MF	WF	WF	EST-GEN-SUL-DOX-TET-CLO-NAL-CIP
7D	*stx1*	MF	MF	WF	AMO-AMP-EST-SUL-DOX-TET-CLO-NAL
7E	*stx1*	WF	NF	NF	AMO-AMP-EST-SUL-DOX-TET-CLO
8A	*stx1*, *stx2*	WF	WF	WF	AMO-AMP-CFZ-CAZ-ERI-EST-DOX-TET-CLO
8B	*stx2*	FM	WF	WF	AMO-AMP-ERI-EST-DOX-TET-CLO-NAL
9A	*stx2*	WF	WF	WF	AMO-AMP-EST-SUL-DOX-TET-CLO-NAL-CIP
9C	*stx1*, *stx2*	WF	MF	SF	CAZ
10C	*stx1*	WF	WF	WF	CFZ-ERI-SUL-DOX-TET-CLO-NAL
11A	*stx2*	MF	MF	SF	AMO-AMP-EST-SUL-DOX-TET-CLO-NAL
11D	*stx1*, *stx2*	MF	MF	WF	AMO-AMP-EST-SUL-DOX-TET-CLO-NAL
12A	*stx1*	WF	MF	SF	AMO-AMP-ERI
12D	*stx1*	WF	WF	WF	AMO-AMP-ERI-SUL

NF = non-biofilm forming; WF = weakly biofilm forming; MF= moderate biofilm former; SF= strong biofilm former. AMO = amoxicillin, AMP = ampicillin, CFZ = cefazolin, CAZ = ceftazidime, ERI = erythromycin, EST = streptomycin, GEN = gentamicin, SUL = Sulfonamide, DOX = doxycycline, TET = tetracycline, CLO = chloramphenicol, NAL = nalidixic acid, CIP = ciprofloxacin.

## Data Availability

The raw data supporting the conclusions of this article will be made available by the authors on request.
